# An unusual extranodal natural killer/T-cell lymphoma presenting as chronic laryngitis: A case report

**DOI:** 10.1097/MD.0000000000026314

**Published:** 2021-06-25

**Authors:** Julio Cruz, Daniela Vargas, Annelisse Goecke, Maria Luisa Molina

**Affiliations:** Service of Rheumatology, Hospital Clínico de la Universidad de Chile, Santiago de Chile.

**Keywords:** chronic laryngitis, extranodal natural killer/T-cell lymphoma

## Abstract

**Rationale:**

Nasal-type, extranodal natural killer (NK)/T-cell lymphoma is a rare lymphoma. The tumor usually shows ulcerative and necrotic lesions in the nasal cavities and sinuses. Tissue involvement outside the nasal cavity is uncommon.

**Patient concern:**

We describe a 30-year-old man with a 2-month history of hoarseness, weight loss, and dyspnea.

**Diagnosis:**

Magnetic resonance image (MRI) showed edema of the larynx with obliteration of the airway. Laryngoscopic examination described necrotic tissue in the glottis and larynx. The biopsy showed chronic, necrotizing laryngitis, with no granulomas, vasculitis, or atypical cells. The immunologic and microbiologic study was negative. Later, after immunosuppressive therapy, the patient presented erythema and diffuse enlargement of the right arm. MRI showed myositis of the biceps and brachial muscles. Infection was rule out, and direct microscopy showed an extensive muscle infiltration by mononuclear cells and abundant mitosis. Immunohistochemistry was positive for CD3, CD8, Ki 67 (90%), and CD56 compatible with extranodal NK/T cell lymphoma.

**Interventions:**

The patient initially received immunosuppression treatments (corticoids, cyclofosfamide, and Rituximab) with relapsing episodes. When lymphoma was diagnosed, chemotherapy was started.

**Outcomes:**

The patient died during chemotherapy.

**Lessons:**

Nasal-type, extranodal NK/T-cell lymphoma should be suspected even when there are no classical findings of neoplasms on histology. Immunohistochemistry is mandatory to rule it out.

## 1 Introduction

Nasal-type, extranodal natural killer (NK)/T-cell lymphoma is a rare lymphoma accounting for only 5% to 10% of non-Hodgkin Lymphoma in Asian and American communities.^[1]^ It can occur at any age but mainly affects subjects during the fourth and fifth decades, predominantly men. It is a clinical entity comprising ulcerative and necrotic lesions preferentially arising in the nasal cavities and sinuses. Other locations occurring outside the nasal cavity are uncommon and usually highly aggressive, with short survival and inadequate response to therapy. One of these rare locations reported is the larynx. However, other locations, such as muscle involvement, have been sporadically reported in the literature.^[2,3]^ Here, we present a 30-year-old man with extranodal NK/T-cell lymphoma of the larynx at presentation and subsequent brachial muscle involvement. Patient informed consent at the time of the final diagnosis was obtained for publication of this case report.

## 2 Case report

A 30-year-old man with a 2-month history of hoarseness, weight loss, and fever received multiples courses of antibiotics and corticosteroids with partial response, developing dyspnea. The patient was hospitalized for further study. A contrast-enhanced computer tomography (CT) of the head and neck showed edema of the larynx without compromising other structures or adenopathies. Direct laryngoscopy exploration showed necrotic tissue and ulcers at the larynx and glottic region, with loss of the normal anatomy of the larynx. A first biopsy described chronic laryngitis with ulcers and unspecific granulation tissue. A microbiologic study, including TB, and the autoimmune study were negative. The patient received antibiotics and corticosteroids to be readmitted to the hospital 1 month later because of fever and dyspnea, requiring a tracheostomy.

A new CT scan showed edema of the larynx with obliteration of the airway. A laryngoscopic examination showed necrotic tissue in the glottis and larynx, and the biopsy informed necrotic tissue with ulcers and granulation in these structures. The immunologic and microbiologic study was negative again, and the patient received cyclofosfamide and corticosteroids. Later on, the patient was hospitalized a few more times for tracheobronchitis, developing severe supraglottic edema and a swallowing disorder.

**Figure 1:**
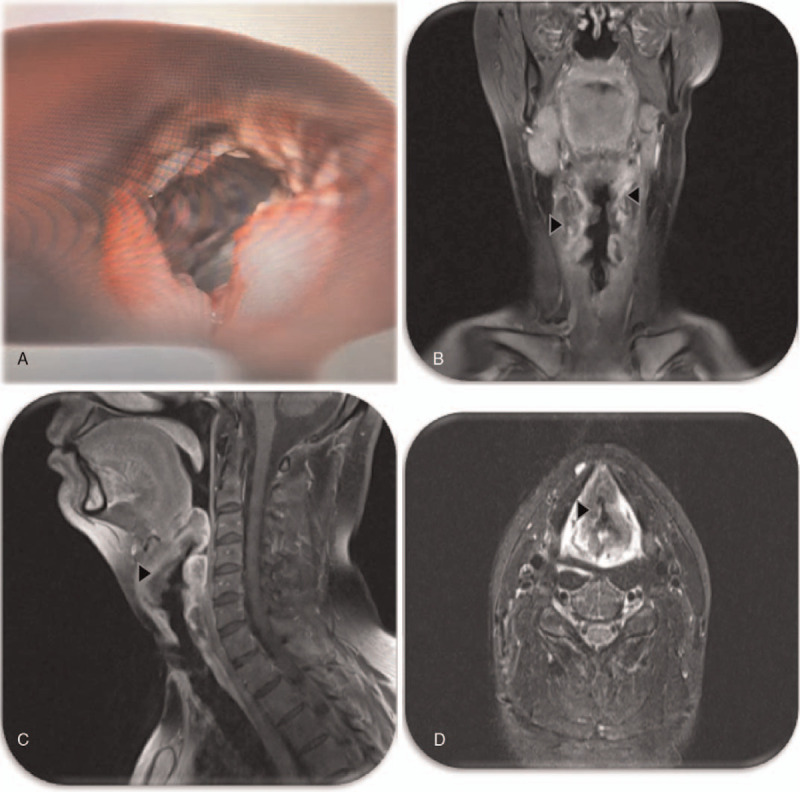
(A) Direct laryngoscopy examination with ulcerative and necrotic lesions at the epiglottis and larynx and loss of the larynx's structure. (B-D) MRI of the neck with an inflammatory compromise of the glottis and epiglottis partially obliterating the airway (arrow head).

The patient was finally admitted to our hospital 4 months later. Laboratory exams showed normal white blood cells, erythrocyte sedimentation rate (16), C reactive protein (64, N < 10), procalcitonin (<0.05), lactate dehydrogenase, renal and hepatic function, and negative microbiologic study (blood, urine, and tracheal secretion cultures, and; markers of hepatitis B virus, hepatitis C virus, human immunodeficiency virus, and syphilis). Immunologic tests such as antinuclear antibody, extractable nuclear antibodies, Anti double stranded DNA, anti-myeloperoxidase, anti-PR3, and C3 and C4 complement were all negative or normal. A direct laryngoscopy examination identified ulcerative and necrotic lesions at the epiglottis and larynx, with loss of the larynx's structure (Fig. 1A). Biopsy specimen and microscopic examination revealed chronic, necrotizing laryngitis, with no granulomas, vasculitis, or atypical cells. Koch culture and TB polymerase chain reaction of tissue samples were negative. Magnetic resonance image (MRI) of the neck demonstrated inflammatory compromise of the glottis and epiglottis partially obliterating the airway (Fig. 1 B-D). Therefore, the patient received 3 boluses of 1 gram of methylprednisolone and started Rituximab (1 gram). A week later, before discharged from the hospital, the patient had normalized inflammatory parameters, and at laryngoscopy, the inflammation and necrotic ulcers at the larynx were in regression.

**Figure 2:**
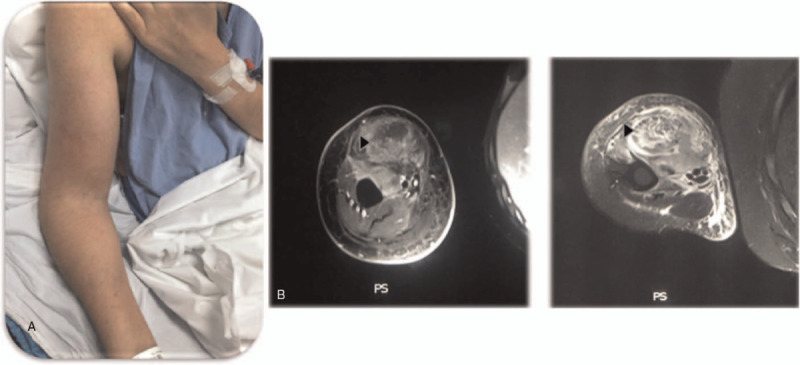
(A) Photograph of the right arm with erythema and diffuse enlargement. (B) MRI of the right arm demonstrating muscle inflammation of the biceps and brachial muscles. (C) Optic microscopy and immunohistochemistry of muscle biopsy.

A month later, the patient presented with a new episode of tracheobronchitis, fever, erythema, and diffuse enlargement of the right arm. MRI showed myositis of the biceps and brachial muscles (Fig. 2 A-B). Abiopsy of the affected biceps was performed. Infection was rule out, and direct microscopy showed an extensive muscle infiltration by mononuclear cells and abundant mitosis. Immunohistochemistry was positive for CD3, CD8, Ki 67 (90%), and CD56 compatible with extranodal Natural killer / T cell lymphoma (Fig. 2C). The previous tracheal biopsy was revised and showed the same results described for muscle biopsy.

The patient received chemotherapy, 6 cycles of cyclophosphamide, hydroxydaunorubicin, oncovine (vincristine), prednisone protocol followed by radiotherapy. Unfortunately, however, the disease was chemoradiotherapy resistant, and he died 9 months after the final diagnosis.

## 3 Discussion

NK / T-cell Lymphoma of the larynx without other involvement is a very uncommon form of presentation, reported in the literature as case reports. In most of these reports, the differential diagnosis is made with ANCA-associated vasculitis, preferentially granulomatosis with polyangiitis (GPA), where the larynx involvement can be the initial manifestation in 1% to 2% of cases. However, in GPA, the larynx involvement is characteristically subglottic with circumferential narrowing. Furthermore, C-ANCA and anti-PR3 antibodies only occur in 47% in the localized forms of GPA. Histopathological finding in the ear, nose, throat area are often nonspecific, where granulomas, vasculitis, and necrosis can be found in only 16% of cases.^[4–6]^

Another important diagnosys to rule out is laryngeal tuberculosis (TB). The larynx involvement is described in 1% of TB patients. Almost always is associated with pulmonary infection and only in 15% of cases is the only manifestation. The most frequently affected area is the true vocal cord, followed by the false vocal cord, epiglottis, arytenoids, and posterior commissure. Sputum smear and cultures are positive only in 40% to 60% of cases, being histology and TB polymerase chain reaction the diagnostic tool of preference where almost 90% of cases can be confirmed. The most common finding in histology are granulomas and ulcerative lesions.^[7–9]^

The prognosis of extranodal NK/T-cell lymphoma depends on the localization. The cumulative probability of survival in the nasal type presentation at 5 years is 37.9% to 45.3%, being worst in presentations occurring outside the nasal cavity.^[10]^ Here we report for the first time a case of extranodal NK/T-cell lymphoma with right upper limb compromise presenting as a brachial and biceps myositis. As far as we know, there are 2 reports in the literature of this type of lymphoma presenting as myositis. Those cases describe a phlegmonous and granulomatous myositis of the lower limb. In one of the reports mentioned, the patient was young as in our case, even though this type of cancer is more common in elderly patients. In both cases, patients died months after initiating therapy, denoting the aggressiveness and refractoriness to therapy of this type of presentation.^[11,12]^

Another significant similarity of the cases reported in the literature with the present report is that the diagnosis was made only after repeated biopsies. Unfortunately, the rate of misdiagnosis described for these lymphomas is up to 70% on the first biopsy, and 3 or more biopsies are needed in 22.5% of cases to reach the correct diagnosis. Perhaps because these tumors often have a pattern of angiocentric growth, causing vascular occlusion and massive tissue necrosis, which makes its interpretation challenging. Indeed, the final histological diagnosis required in all cases, including ours, the immunohistochemistry analisys specific for the Extranodal NK / T-cell lymphoma: cells positive for granzyme B, CD3, CD45RO, and CD56, and negative for CD20 and ALK1.^[2]^

Finally, in the nasal type, extranodal NK/T-cell lymphoma B-symptoms are present in only 50% of cases, and lactate dehydrogenase can be normal in 70%, making the diagnosis difficult when the patient had an atypical presentation without nasal involvement. For this reason, it is essential to emphasize the requirement for repeated biopsy when this lymphoma is suspected and the necessity of immunohistochemistry even though the classical findings of neoplasm are not present at microscopy.^[2]^

## Author contributions

**Conceptualization:** Annelisse Goecke, Maria Molina.

**Data curation:** Julio Cruz, Daniela Vargas.

**Formal analysis:** Annelisse Goecke, Maria Molina.

**Investigation:** Maria Molina.

**Supervision:** Annelisse Goecke.

**Validation:** Maria Molina.

**Visualization:** Maria Molina.

**Writing – original draft:** Maria Molina.

**Writing – review & editing:** Maria Molina.
